# Feasibility of a cohort study on health risks caused by occupational exposure to radiofrequency electromagnetic fields

**DOI:** 10.1186/1476-069X-8-23

**Published:** 2009-05-29

**Authors:** Jürgen Breckenkamp, Gabriele Berg-Beckhoff, Eva Münster, Joachim Schüz, Brigitte Schlehofer, Jürgen Wahrendorf, Maria Blettner

**Affiliations:** 1 Department of Epidemiology and International Public Health, Faculty of Health Sciences, Bielefeld University, Universitätsstraße 25, 33615 Bielefeld, Germany; 2 Institute of Occupational, Social, and Environmental Medicine, Johannes Gutenberg-University of Mainz, Obere Zahlbacher Straße 67, 55131 Mainz, Germany; 3 Institute of Cancer Epidemiology, Danish Cancer Society, Strandboulevarden 49, DK-2100 Copenhagen, Denmark; 4 Unit of Environmental Epidemiology, German Cancer Research Center, Im Neuenheimer Feld 280, 69120 Heidelberg, Germany; 5 Institute of Medical Biostatistics, Epidemiology, and Informatics, Johannes-Gutenberg-University of Mainz, Obere Zahlbacher Straße 69, 55131 Mainz, Germany

## Abstract

**Background:**

The aim of this study was to examine the feasibility of performing a cohort study on health risks from occupational exposure to radiofrequency electromagnetic fields (RF-EMF) in Germany.

**Methods:**

A set of criteria was developed to evaluate the feasibility of such a cohort study. The criteria aimed at conditions of exposure and exposure assessment (level, duration, preferably on an individual basis), the possibility to assemble a cohort and the feasibility of ascertaining various disease endpoints.

**Results:**

Twenty occupational settings with workers potentially exposed to RF-EMF and, in addition, a cohort of amateur radio operators were considered. Based on expert ratings, literature reviews and our set of predefined criteria, three of the cohorts were identified as promising for further evaluation: the personnel (technicians) of medium/short wave broadcasting stations, amateur radio operators, and workers on dielectric heat sealers. After further analyses, the cohort of workers on dielectric heat sealers seems not to be feasible due to the small number of exposed workers available and to the difficulty of assessing exposure (exposure depends heavily on the respective working process and mixture of exposures, e.g. plastic vapours), although exposure was highest in this occupational setting. The advantage of the cohort of amateur radio operators was the large number of persons it includes, while the advantage of the cohort of personnel working at broadcasting stations was the quality of retrospective exposure assessment. However, in the cohort of amateur radio operators the exposure assessment was limited, and the cohort of technicians was hampered by the small number of persons working in this profession.

**Conclusion:**

The majority of occupational groups exposed to RF-EMF are not practicable for setting up an occupational cohort study due to the small numbers of exposed subjects or due to exposure levels being only marginally higher than those of the general public.

## Background

The widespread use of cordless and cellular phones led to a rapid increase in the number of persons exposed to radiofrequency electromagnetic fields (RF-EMF), accompanied by concerns and fears concerning possible adverse health effects of RF-EMF exposure being widely raised. In total, 48% of citizens of the Europe Union (25 member states) are very much or fairly concerned about potential health risks of electromagnetic fields [1]. The concern and fears in the population have be taken seriously in research, but further research on potential health effects should be conducted. Neither research on new wireless devices like mobile communication, nor research on occupational exposure is sufficient to rule out chronic health effects from low exposures. Due to the widespread use, an undetected RF health effect might become a serious public health problem before environmental health authorities can respond.

Although there are currently many studies available on the health risks of mobile phone use, the evidence is inconclusive [2-9]. A serious problem in the current studies on mobile phone use is the relatively short latency period. Up to now, data from only two cohort studies are available [10,11]. The update of the Danish cohort study with an average follow-up time of 8.5 years revealed no increased cancer risk [12]. In the case-control studies on this topic, recall bias and selection bias are a major concern.

It is of public health interest to get valid estimates of RF-EMF disease risks, emitted from mobile phone handsets and masts as soon as possible. An alternative would be to investigate other RF-EMF exposures with longer exposure times, for example occupational RF-EMF exposure. So far, only a few epidemiological cohort studies have investigated the effects of RF-EMF on health in occupational settings or during leisure time (amateur radio operators) [13-22]. In these studies, total mortality, partially differentiated by important groups of diseases and/or cancer incidence or cancer mortality, especially brain tumour and leukaemia, were defined as outcomes. One common problem in all these studies is the assessment of exposure [23]. In most occupational cohort studies it is exceedingly difficult or too expensive to determine the individual exposure. On the other hand, a large number of subjects is needed to achieve a sufficient statistical power to detect presumable small health risks.

As case-control studies generally focus on fewer numbers of subjects than cohort studies, the assessment of occupational RF-EMF exposure seems to be more feasible. Hence, the analysis of the association between RF-EMF exposure and health risks might be more appropriately done using case-control study designs. So far, two case-control studies on brain tumour and occupational exposure of RF-EMF were conducted. In one of them, a nested case-control study, the complete occupational histories were obtained from personnel records of each study subject including job title and starting and ending dates [24]. In a second interview based study, a more specific, activity related RF-EMF exposure during work and leisure time was estimated using an activity exposure matrix [25]. Even though positive significant associations have been reported in one of these occupational case-control studies, the overall picture is far from being clear.

Given the well-known problems in occupational case-control studies, the aim of the present study was to examine the feasibility of a cohort study on health risks due to radiofrequency exposure except mobile phone use. This study design was chosen due to the advantage of a cohort study: prospective assessment of exposure and several diseases as outcome.

A major objective for the feasibility study was to investigate health effects in a group of persons in which a reasonable exposure assessment was feasible, and in which a potential for some high and long-lasting exposure was present.

## Methods

Firstly, different occupational groups and amateur radio operators were considered as potential cohorts with high and long-lasting exposure to RF-EMF. (In the following text the term occupational groups/cohorts is used for both the occupational cohorts and the cohort of amateur radio operators). Information about possibly exposed occupational groups was obtained from different professional associations, visits of industry sites and contacts with committees and administrative bodies. We performed a literature review including published papers and technical reports. We searched for studies on the subject "health risk by exposure to radiofrequency electromagnetic fields". The search was performed using the literature data bases PubMed and CancerLit. Different search terms were used, among others: "electromagnetic fields", "radiation", "radiation/adverse effects", "cancer" and "cohort study". In PubMed the filter "human" was set. In CancerLit, the filter "non Medline" was additionally used. Furthermore, a search with given medical subject headings (MeSH) was carried out in PubMed, i.e. "electromagnetic fields/adverse effects", "radiation non-ionizing/adverse effects". For this search the methodical filter "cohort study" was additionally used. All searches were restricted to publications in the English and the German language. Further information is given in [23]. In addition, technical reports [26-31] were searched for on the Internet. Internal papers in the field of occupational medicine were also reviewed. The purpose of this was to identify jobs and occupational settings in which exposure to RF-EMF may play a major role.

### Definition of outcomes

Possible outcomes of the cohort study were identified by reviewing the literature. In a first step, we compiled a list of all diseases mentioned as outcomes in these papers. Morbidity studies as well as mortality studies were considered. In a further step it was decided that mainly mortality should be considered as an outcome, because then a retrospective part of the cohort study would be possible in Germany. Thus, mortality from cancer, cardiovascular diseases, and neurodegenerative diseases were determined as possible outcomes for the cohort study.

### Criteria for assessment

After identifying potential occupational cohorts, we checked whether each of them fulfilled our pre-defined set of criteria.

Four criteria were used:

#### Criteria for RF-EMF exposure

Subjects in an occupational cohort have to be exposed continuously as well as over a long period of time of ten years or more, and the exposure has to be higher than that in the general population. In addition, the duration of employment as well as information about the affiliation to an occupational group must be available for at least 90 to 95% of all members of the cohort.

#### Criteria for exposure assessment

Prospective exposure estimates should be possible on an individual level. A retrospective estimate of exposure should be possible at least on an aggregated level by using a job-exposure-matrix.

#### Criteria for assembling the cohort

A well defined group of persons needs to be available. It must be possible to choose an unselected cohort from personnel records of the employers, public authorities or companies. Demographic variables must be available from documents of the employers, and should retrospectively be available for at least 5 or 10 years. Cohorts in large companies or public authorities are of special interest, as it would be difficult to include a large number of small factories or companies for the study. Representatives of employees and the management of the company must be willing to endorse the study. Important parameters were also the total numbers of persons employed in positions exposed to RF-EMF.

#### Criteria for the follow-up

A follow-up of preferably all members of the cohort is needed. As a cohort study with mortality as outcome was planned, follow-up can be easily carried out if the addresses (even former) of the cohort members are available. In this case the follow-up can be done through registration offices and public health authorities. Therefore cohorts could only be eligible if the addresses of cohort members were available.

### Calculation of expected cases

To calculate the power of a planned cohort, assumptions have to be made about the size of the cohort in terms of person years, the age distribution and the expected relative risk for exposed persons. The following assumptions were used for the power calculation: lag period = 5 years, loss to follow-up = 5%, age of study population 20 to 59 years with a uniform age distribution. Table 1 shows the expected number of deaths under different conditions of follow-up. The numbers of deaths in bold are needed if a significance level of 0.05 and a power of 80% is required. It should be noted that doubling the cohort size means that the numbers of expected cases are doubled, while doubling the observation time may increase the number of expected cases substantially (table 1) as the cohort is aging. 5000 subjects and a 30-year follow-up are needed to reject the hypothesis of "no increased risk" (RR = 1) (number of cases >= 30), if the true relative risk is greater or equal 1.5.

**Table 1:** Expected number of deaths and its dependency on cohort size and follow-up

Cohort	N = 5,000				N = 10,000			
Follow-up (in years)	15		30		15		30	

	female	male	female	male	female	Male	female	male

Person years	45 970	44 464	105 356	96 535	91 939	88 929	210 712	193 069

Total mortality	**180**	**347**	**1 115**	**1 624**	**361**	**695**	**2 231**	**3 248**

CHD**	**53**	**132**	**488**	**709**	**107**	**264**	**977**	**1 418**

CHD without ischemia	12	25	**108**	**123**	25	**50**	**216**	**246**

accidents	12	**33**	**50**	**100**	25	**66**	**100**	**207**

Suicide	6	14	21	**39**	13	29	**42**	**78**

All cancers	**74**	**102**	**326**	**417**	**148**	**204**	**652**	**835**

Breast	18	0	**58**	0	**36**	0	**117**	1

Nervous system	3	4	22	25	7	9	**44**	**51**

Lymphatic tissue	2	3	10	14	5	7	21	28

Leukemia	2	2	9	11	4	5	19	23

Brain	1	2	5	6	3	5	9	12

* Bold marked figures show the expected number of deaths, fulfilling the given criteria of a significance level of 0.05, a statistical power of 80%, an estimated RR of 1.50 to reject the null-hypothesis

For example, if we are interested in brain cancer, we need 10,000 subjects and a 30-year follow-up to reject a RR = 1.0 (number of cases >= 9), if the true RR is greater or equal 2.0, which is given analyzing brain cancer in females (n = 9) and males (n = 12).

The numbers in table 1 demonstrate that for a prospective cohort study, meaningful results can not be expected within a few years. For this reason, only a retrospective (historical) cohort study seems to be appropriate.

## Results

Twenty occupational settings and amateur radio operators were considered as potentially exposed to RF-EMF (table 2). Exposure levels of all potential cohorts were rated by experts, e.g. occupational hygienists. Most of the experts' ratings were based on results of measurements, performed in different industries, mainly done in the frame of preventive actions to avoid work-related health hazards. At least two experts were asked for each of the occupational settings.

**Table 2:** Occupational cohorts initially defined as exposed*

*Transmitters and radar*	*Industry* *(cohort working with...)*	*Others*
Captain in inland water transport and working in a sluice (1)	Deep drawing machine (1,2,3)	Assistant medical technician, physiotherapist, physician assistant in hyperthermia, diathermy (2)
	**RF plastic welding**	
Airport workers (1)	WIG-welding (1)	Cashier (EAS, anti-theft-device) (2)
Telecommunication technicians (antenna tests) (1,2)	Blister packaging (2,3)	
**Amateur radio operators**	Chip production (1)	
Telecommunication technicians (general) (1)	Dielectric vulcanizing (2)	
Fire brigade, emergency medical services, police (1,2)	High frequency generator (1)	
Roofer, work on scaffolding, chimney sweeper (1)	Induction machines (2)	
**Workers on short- and medium wave transmitters**	High frequency dryer (2)	
RF-research institutes (2)	Gluing press (2)	

* Bold text identifies cohorts that were investigated in more detail; numbers in brackets provide the reasons why this particular potential cohort was excluded from the detailed investigation

1 = Not exposed or low exposure levels

2 = Small number of exposed subjects, establishing a cohort not possible

3 = Automated and/or shielded working processes

Eighteen of the twenty-one situations described in table 2 were not further considered for a cohort study for one or more of the following reasons:

1. Exposure to RF-EMF rare or at very low level: e.g. for captains and boat personnel and persons working in a lock, exposure to RF emitted by radio communication does not play a major role. An exposure to microwave emitted by radar is probably very low if present at all, as the position of radar equipment is on the roof of the ship and rather far away from the working position.

2. Small number of exposed subjects: e.g. firms where gluing presses are used. Generally only very few persons are highly exposed in these firms. Assembling a cohort seems to be impracticable.

3. Automated and shielded working processes: e. g. blister packaging, is an automated and shielded process. Maintenance of the devices only takes place when the machine is switched off (see table 2).

Comments and conclusions for exclusion are presented in table 3. Only three groups of persons were further considered after these considerations: (1) Personnel of medium/short wave broadcasting stations, (2) amateur radio operators, and (3) workers on dielectric heat sealers.

**Table 3:** Selection of potential study population for an occupational RF-EMF cohort and the explanation for not being considered in the ongoing feasibility study

Airport workers (working on the apron of the airfield)	Study population:	1000 workers
	Comment:	In the past apron personnel was highly exposed, because nose radars of airplanes were not switched off. According to experts nowadays the personnel is not exposed.
	Conclusion:	Not exposed
Assistant medical technician, Physiotherapist, Physician assistant in hyperthermia/diathermia	Study population:	Few persons, if any
		Frequent hyperthermia- and diathermia-therapy in the 60^th ^and 70^th^. Exposure in some cases above threshold. Since then the number of therapies decreased, because it is not reimbursed through the public or private health insurance. Today this therapy does not play a role in Germany.
	Conclusion:	Establishing a cohort with retrospective data not possible

Blister packaging	Study population:	One maintenance technician per Company
	Comment:	Shielded and automated process and therewith low exposure level.
	Conclusion:	Not exposed or only a few number of possibly exposed persons

Captain in inland water transport and working in a sluice	Study population:	3200 captains and 700 lock keepers
	Comment:	According to experts the exposure to radio frequencies does not play a role. An exposure to radar is questionable due to technical reasons: radar units are mounted on the roof of the ships.
	Conclusion:	Not exposed or low exposure levels

Cashier (EAS, anti- theft-device	Study population:	?
	Comments:	Many systems using different electromagnetic fields are in use. Radiofrequency systems are only a part of it. The cohort is limited by short duration of employment and low mean age of the cashiers.
	Conclusion:	Establishing of a long term exposed cohort not possible

Chip production	Study population:	?
	Comment:	Closed systems (vaporization is a shielded process)
	Conclusion:	Not exposed

Deep drawing Machines	Study population:	One person per unit
	Comment:	Most often machines work with hot air and shrink films. Only few machines work with radiofrequency. Normally these machines are shielded, automated working process, and are switched off for maintenance work. Work is done by one person.
	Conclusion:	Not exposed or only few possibly exposed persons

Dielectric Vulcanizing	Study population:	?
	Comment:	No precise information on the exposure was available
	Conclusion:	Small number of exposed persons is expected

Fire brigade, emergency medical services, police	Study population:	?
	Comment:	Exposure from radio frequency is low because external vehicle antennas are used – exposure to radar (police officers) is low, hand hold devices are rarely used.
	Conclusion:	Low exposure levels at time of the study

Gluing press	Study population	One carpenter per specialized company
	Comment	There might be some highly exposed workplaces in specialized small companies.
	Conclusion:	Small number of exposed persons

High frequency- induction machines	Study population:	About 30 employees per hardening shop
	Comment:	Number of exposed employees unknown. Most equipments work in the frequency range from 3 to 10 KHz. To avoid radio interferences, these machines are shielded.
	Conclusion:	Small number of exposed persons

High frequency Dryer	Study population:	?
	Comment:	Operating personnel will leave the construction site or e.g. the garret.
	Conclusion:	Small number of exposed persons

Production of high Frequency generators	Study population:	100 persons
	Comment:	Only few high frequency generators are produced in Germany. Number of possibly exposed employees is small.
	Conclusion:	Small number of exposed persons

Radiofrequency- Research Institutes	Study population:	150 persons
	Comment:	Unknown, how many persons are actually exposed – Exposed personnel possible exposed to different frequencies
	Conclusion:	Small number of exposed persons

Telecommunication technicians (antenna tests)	Study population:	Three or four companies, number of technicians unknown subgroup of "telecommunication technicians (general)".
	Comment:	During maintenance of antennas they are switched off. Only employees testing antennas are potentially exposed. But tests of antennas take place in shielded rooms.
	Conclusion:	Not exposed

Telecommunication technicians (general)	Study population:	30,000 persons
	Comment:	1) Most of maintenance work on mobile phone antennas can be done electronically at the socket of the masts. 2) Possibly exposed during replacement of obstruction lights – but amplitude modulated transmitter stations are switched off in this case – number of concerned technicians unknown.
	Conclusion:	Not exposed or only few possibly exposed persons

Roofer, work on scaffolding, chimney sweeper	Study population:	?
	Comment:	Exposed when working near to antennas, exposure is high but seldom. Retrospective exposure conditions can not be obtained.
	Conclusion:	Not exposed or low exposure level

WIG-welding	Study population:	?
	Comment:	Nearly all welding techniques (including so called high frequency welding) work with low frequencies (up to 2000 Hz) – some techniques use radio frequency ignition – in this case workers are exposed momentarily.
	Conclusion:	Not exposed or low exposure level

### Personnel of medium/short wave broadcasting stations

At broadcasting stations about 200 to 250 employees (technicians) are potentially exposed to radiofrequencies from the antennas (medium wave: 526.5 kHz – 1.6065 MHz, bandwidth 9 kHz, or short wave: 3.4 MHz – 26.0 MHz, bandwidth 5 kHz). Personnel are employed only on stations with a transmitting power of ≥ 100 kW. In Germany 20 of 29 broadcasting stations operate at this power level. The employees in these workplaces (e.g. mechanical workshop) are continuously exposed and the duration of the exposure corresponds to the total working time.

The exact determination of the current individual exposure can be obtained by measurements. In addition, it is possible to obtain an estimation of past exposure from computer simulations basing on current exposure. This is important because the antennas were previously operated partly with a higher transmitting power and other modulation procedures. The operating conditions (transmitting power and field-strength) of the amplitude modulated medium wave antennas can be traced back quite well over approximately the last 20 years.

The strengths of this cohort would be the readiness for co-operation and support of the project, the measurable and/or valid estimation of exposure, the almost daily exposure over a long time of the working life, the relatively high constancy of the cohort and the good accessibility. Furthermore personnel data are retrospectively available for a period of at least 10 years.

The limitations of the study design include the rather low levels of exposure (mechanical workshop, Mühlacker broadcasting station, electrical field: 1.5 V/m, magnetic field: 0.2 A/m), the small cohort size (maximum 250 potentially exposed persons in Germany) and the fact that such a cohort consists exclusively of persons in technical occupations (highly selective group).

### Amateur radio operators

Altogether, 80,000 amateur radio operators are registered in Germany. It is estimated that only two-thirds of them are active.

Radio equipment with a transmitting power of > 10 W is notifiable. The permissible frequencies for amateur radios lie between 2 MHz and about 300 GHz. However, less than 5% of amateur radio operators transmit in the frequency range 900 – 2.200 MHz and above. This is because most amateur radio operators do not possess the necessary technical equipment to transmit within this frequency range.

The average exposure time of an amateur radio operator will rarely exceed the value of 10 h/week, with large individual variation. A high exposure to RF-EMF arises as a result of the adjustment of the antenna or other work on the radio transmitters and radio traffic with an antenna which is installed in the house. Measured exposure values are not available. The whole body exposure, amongst others from antennas installed within the house, can however be comparably high.

#### Advantages

A cohort with a large size can easily be ascertained and, due to the structure of the organization of amateur radio operators, is also retrospectively available for many years. Demographic data of the members are present, also for the past. Long-term exposure is common and varies widely among the individual members. The fluctuation of the membership is small due to the high expenditure involved (examination, costs for radio equipment).

#### Disadvantages

Amateur radio operators are most likely not exposed to RF-EMF on a daily basis. This cohort was comprised of quite a specific study population (technicians and handicapped persons) which can not be easily compared with the general population.

#### Workers on dielectric heat sealers

High frequency dielectric heat sealers are mainly used for welding of plastic products, operate with the industrial frequency of 27.12 MHz and have been used in Germany since the 1960's. At dielectric heat sealers, the workers are mainly occupied with the introduction and removal of the product to be welded. Depending upon the shielding of the electrodes, exposure to different levels of RF-EMF can occur.

Measurements done at the request of the Lower Saxony Ministry of Social Affairs (Niedersächsisches Sozialministerium) and the Regional Office for Ecology (Niedersächsisches Landesamt für Ökologie) in 1996 showed that the majority of plants exceeded the licit exposure range. Discussions held with the Lower Saxony Regional Office for Ecology and the Trade Association for Precision Mechanics and Electro-Technology also confirmed that workers on dielectric heat sealers are highly exposed to RF-EMF.

The availability of information about the number of exposed workers per company was scarce. Interviewed experts of different professional associations and the Lower Saxony Regional Office for Ecology stated that in general these workers are employed for a long time. They also believed that protective clothing was rarely used. The workers are employed in small and medium sized companies.

The disadvantage of this cohort is the small number of exposed workers per company, as this would necessitate contacting a large number of companies. It was also unclear whether appropriate measurement of exposure could be obtained in all these firms. Additionally, several other occupational exposures (plastic vapours, low frequency fields, and noise) can arise. It was also not possible to obtain an estimation of the number of exposed employees due to an incomplete view of the company structures.

Measurements of the Lower Saxony Social Department and of the Trade Association of the Chemical Industry showed that workers on dielectric heat sealers are exposed to considerably higher levels of RF-EMF than the general population. The fact that workers are continuously employed at the same company for long periods of time and that they work on a regular daily basis on these machines support the classification of the long exposure duration as suitable (see additional file 1).

The exposure of the cohort of workers on dielectric heat sealers is high; however, ascertainment of the cohort is rather difficult. The strength of the cohort of amateur radio operators is the large number of persons; however, exposure assessment seems to be difficult. The strength of the cohort of technicians is the quality of retrospective exposure assessment, but epidemiological research is hampered by the small number of persons working in this profession.

## Discussion

Our aim was to investigate the possible health effects of RF-EMF exposure in a large retrospective occupational cohort. We therefore considered different groups of persons potentially exposed to RF-EMF, identified by literature search and knowledge about potential exposures, and explored whether it would be feasible to set up epidemiologic research in these groups. Most of the eligible occupational groups can not be realistically used for a cohort study as the numbers of exposed subjects are small, or exposure levels are only marginally higher than those of the general public. We identified three promising groups for a more detailed assessment: professionals in broadcasting stations, amateur radio operators and workers on dielectric heat sealers. However, further inspection of those groups revealed major obstacles. The implementation of digital broadcasting and the intention to switch-off all analogue broadcasting frequencies in Germany by 2010 makes a cohort study with technicians of medium and short wave broadcasting stations seemingly obsolete. A historical cohort has little power as the total number of persons working in this field is low. For amateur radio operators the average exposure is rather low. Moreover, an exposure assessment is difficult and seems to be feasible only by using a questionnaire for each individual. Workers on dielectric heat sealers work in many small firms, which are spread all over Germany. They have a mixture of exposures (e.g. vapours, low frequency fields) and different operational procedures, depending on the current manufacturing process (see additional file 1: Summary of results). The result of our feasibility study was that we recommended not performing a cohort study to investigate occupational RF-EMF exposure at that time.

Our results are based on the situation in Germany and are only partly transferable to other countries. For example most German firms which use dielectric heat sealers have gone abroad (to countries with less labour protection) because they could not comply with the German labour protection regulations, which are stricter than the ICNIRP's (International Commission on Non-Ionizing Radiation Protection) reference exposure levels [32,33]. Hence the number of workers at dielectric heat sealers might be substantially higher in other countries. Our concern to perform a cohort study among amateur radio operators may, however be true for other countries. As broadcast tower technicians appear to be a small occupational group in many countries, huge international efforts would be needed to establish a large enough cohort of workers.

## Conclusion

The aim of our study was to identify cohorts of subjects occupationally exposed to RF-EMF and to investigate the feasibility of conducting such a cohort study in Germany. We identified three cohorts (technicians of medium and short wave broadcasting stations, amateur radio operators, workers on dielectric heat sealers), in which the investigation of RF-EMF-associated health risks is, in principle, feasible. The conduction of a cohort study with persons exposed to RF-EMF poses a number of methodological problems that seem difficult to overcome. In the meantime, prospective cohort studies of mobile phone users became an option and have been started in some countries.

## Abbreviations

A/m: ampere per meter; GHz: gigahertz; h/week: hours per week; kHz: kilohertz; kW: kilowatt; V/m: volt per meter; MeSH: medical subjects headings; MHz: megahertz; RF: radiofrequencies; RF-EMF: radiofrequency electromagnetic fields; W: watt.

## Competing interests

The authors declare that they have no competing interests.

## Authors' contributions

MB, GBB, BS, JW and JS conceptualised the study and developed the study protocol. JB and EM were responsible for the conduction of the study. GBB and JB wrote the initial draft of the paper, which was subsequently modified in discussions with all authors. JB is the guarantor of the work. All authors read and approved the final manuscript.

## Supplementary Material

Additional file 1 **Summary of results**. The table summarizes the advantages and disadvantages of the three selected potential cohorts. Click here for file
