# Supplementary material for: Feasibility of a cohort study on health risks caused by occupational exposure to radiofrequency electromagnetic fields
Source: Environ Health. 2009 May 29;8:23. doi: 10.1186/1476-069X-8-23 (PMC2694786; doi:10.1186/1476-069X-8-23)
Supplement: Additional file 1 — Summary of results. The table summarizes the advantages and disadvantages of the three selected potential cohorts. [file 1476-069X-8-23-S1.doc]

## Table S1 - Summary of results

|  | | Employees on medium wave and short wave broadcasting stations | Amateur radio operators | Workers on RF-dielectric heat sealers |
| --- | --- | --- | --- | --- |
| Exposure conditions | Regular exposure | yes | no | yes |
|  | Level of exposure | low  (1.5 V/m) | medium  (according the German Amateur Radio Club in some cases – e.g. in house antenna -above German threshold) | high  (partly above German threshold*) |
|  | Duration of exposure | high | high | medium |
|  | Retrospective coverage | very good | possible | possible |
|  | Period of employment | long time | long time | long time |
| Setup of a cohort | Demographic variables | easy access | easy access | accessible |
|  | Retrospective available | easy access | easy access | accessible |
|  | Large companies, - authorities, -organizations | medium-sized organization | large organization | small companies |
|  | Preparedness for cooperation | very good (SWR – South West Radio) | very good (German Amateur Radio Club) | very good (BG - Professional Association)  problematic (companies) |
|  | Expected number of cases | N max. = 250 | N = 50,000 active operators, additionally a higher exposed subgroup should be selected (working with 700 W of transmitting power) | N max. = 1000** |
| Baseline | Questionnaire and medical assessment | possible | possible | possible |
| Follow-up | Retrospective follow-up | possible | possible | difficult |
|  |  | an international cohort is necessary to get sufficient numbers for mortality follow-up | also a prospective part of follow-up is necessary to evaluate exposure | very small numbers for mortality follow-up |
| Conclusion |  | obsolete because of switch-off of all analogue frequencies in 2010 | cohort study feasible in principle | cohort study feasible in principle, but more disadvantages than the other potential cohorts |

* The threshold (26th Ordinance regulating the Federal Pollution Control Law (26. BImSchV 1996)) is oriented to ICNIRP guidelines

** Estimate
